# *Aspergillus flavus* pangenome (AflaPan) uncovers novel aflatoxin and secondary metabolite associated gene clusters

**DOI:** 10.1186/s12870-024-04950-8

**Published:** 2024-05-01

**Authors:** Sunil S. Gangurde, Walid Korani, Prasad Bajaj, Hui Wang, Jake C. Fountain, Gaurav Agarwal, Manish K. Pandey, Hamed K. Abbas, Perng-Kuang Chang, C. Corley Holbrook, Robert C. Kemerait, Rajeev K. Varshney, Bhabesh Dutta, Josh P. Clevenger, Baozhu Guo

**Affiliations:** 1 Department of Plant Pathology, University of Georgia, Tifton, GA 31793 USA; 2 Crop Protection and Management Research Unit, USDA-ARS, Tifton, GA 31793 USA; 3 HudsonAlpha Institute for Biotechnology, Huntsville, AL 35806 USA; 4 International Crop Research Institute for the Semi-Arid Tropics (ICRISAT), Hyderabad, 502324 Telangana India; 5 Department of Plant Pathology, University of Georgia, Griffin, GA 30223 USA; 6 Department of Plant Biology, Michigan State University, East Lansing, MI 48823 USA; 7 Biological Control of Pests Research Unit, USDA-ARS, Stoneville, MS 38776 USA; 8 Southern Regional Research Center, USDA-ARS, New Orleans, LA 70124 USA; 9 WA State Biotechnology Centre, Centre for Crop and Food innovation, Food Futures Institute, Murdoch University, Murdoch, WA 6150 Australia

**Keywords:** Aspergillus flavus, Aflatoxin, Core genome, Accessory genome, Genomic diversity, pan-GWAS

## Abstract

**Background:**

*Aspergillus flavus* is an important agricultural and food safety threat due to its production of carcinogenic aflatoxins. It has high level of genetic diversity that is adapted to various environments. Recently, we reported two reference genomes of *A. flavus* isolates, AF13 (*MAT1-2* and highly aflatoxigenic isolate) and NRRL3357 (*MAT1-1* and moderate aflatoxin producer). Where, an insertion of 310 kb in AF13 included an aflatoxin producing gene bZIP transcription factor, named *atfC*. Observations of significant genomic variants between these isolates of contrasting phenotypes prompted an investigation into variation among other agricultural isolates of *A. flavus* with the goal of discovering novel genes potentially associated with aflatoxin production regulation. Present study was designed with three main objectives: (1) collection of large number of *A. flavus* isolates from diverse sources including maize plants and field soils; (2) whole genome sequencing of collected isolates and development of a pangenome; and (3) pangenome-wide association study (Pan-GWAS) to identify novel secondary metabolite cluster genes.

**Results:**

Pangenome analysis of 346 *A. flavus* isolates identified a total of 17,855 unique orthologous gene clusters, with mere 41% (7,315) core genes and 59% (10,540) accessory genes indicating accumulation of high genomic diversity during domestication. 5,994 orthologous gene clusters in accessory genome not annotated in either the *A. flavus* AF13 or NRRL3357 reference genomes. Pan-genome wide association analysis of the genomic variations identified 391 significant associated pan-genes associated with aflatoxin production. Interestingly, most of the significantly associated pan-genes (94%; 369 associations) belonged to accessory genome indicating that genome expansion has resulted in the incorporation of new genes associated with aflatoxin and other secondary metabolites.

**Conclusion:**

In summary, this study provides complete pangenome framework for the species of *Aspergillus flavus* along with associated genes for pathogen survival and aflatoxin production. The large accessory genome indicated large genome diversity in the species *A. flavus*, however AflaPan is a closed pangenome represents optimum diversity of species *A. flavus*. Most importantly, the newly identified aflatoxin producing gene clusters will be a new source for seeking aflatoxin mitigation strategies and needs new attention in research.

**Supplementary Information:**

The online version contains supplementary material available at 10.1186/s12870-024-04950-8.

## Introduction

*Aspergillus flavus* is an opportunistic saprophyte, infects varieties of crops such as maize and peanuts both pre- and postharvest [1]. These fungi also produce secondary metabolites including aflatoxins which contaminate the grains and are toxic and carcinogenic leading to inducing acute and chronic health issues in both humans and animals [2]. In addition to aflatoxins, *A. flavus* also produces cyclopiazonic acid (CPA), which harms liver, kidneys, and gastrointestinal tract [3]. Because of its harmful impact on human health, the aflatoxin is considered as “hidden and slow poison” and the research community is trying its best to managing through this menace through a combination of improved genetics and post-harvest (farm to industry) management on one side while understanding the reason of compulsion for pathogen to produce toxin on the other side [4]. Aflatoxin contamination is an annual issue in the southern United States and throughout the world, but occasional outbreaks happen in the “cooler areas” like mid-western Corn Belt of the United States [5].

*Aspergillus flavus* belongs to *Aspergillus* section *Flavi*, which splits into eight clades and currently contains 33 species. Of these, two species only produce aflatoxin B1 and B2 (*A. pseudotamarii* and *A. togoensis*), and 14 species are able to produce aflatoxins B1, B2, G1 and G2 [6]. Large differences in gene contents can occur among genomes of *A. flavus* isolates, with only a portion of genes being universal, or core, to all genomes. For instance, *A. flavus* and *A. oryzae* possess core identical gene models and sequences, however *A. flavus* is a very devastating fungal pathogen produces aflatoxin, while *A. oryzae* is a beneficial fungus used widely for fermentation in food industries [7, 8]. Interestingly, comparative genomic analysis of these two Aspergilli clearly shows that *A. oryzae* is a domesticated ecotype of wild *A. flavus* [9]. Therefore, a single reference genome could not represent complete diversity of a species [10]. A recent pan-metabolome analysis of 94 *A. flavus* isolates support this with identification of 7821 biosynthetic gene clusters (BGCs) including 25% population specific BGCs and 92 unique BGCs [11].

*A. flavus* isolate NRRL 3357 was used as a reference genome for multiple omics studies [12]. Recently, the NRRL 3357 assembly was updated and re-annotated for eight chromosomes [13]. At the same time [14], reported two new *A. flavus* reference genomes comparatively, which revealed a large insertion potentially contributing to stress tolerance and aflatoxin production in isolate AF13, a *MAT1-2* mating type in comparison with NRRL 3557, a *MAT1-1* mating type. This study between AF13 and NRRL 3357 confirmed that AF13 has an insertion of 310 kb with a bZIP transcription factor named *atfC* [14], which shares homology with *A. flavus* bZIP transcription factor *atfA* [15] and *atfB* [16]. These transcription factors have been shown to regulate the production of aflatoxin and its precursors in response to oxidative stress and to coordinate oxidative stress responsive genes including catalase [17]. If using only the NRRL 3357 reference genome, the presence of this insertion the *atfC* gene would not have been recognized due to reference bias.

Therefore, use of single reference genome, either NRRL 3357 or AF13, hinders the potential of omics studies to fully describe and investigate complex pathways such as aflatoxin or other secondary metabolite biosynthesis in *A. flavus*. It is essential to study the comprehensive genetic diversity of *A. flavus* using a large number of isolates from diverse geographical regions and ecosystems [9]. Pangenomics has emerged as an effective approach to explain the widespread diversity present in a species, and super-pangenome can be used to represent the diversity present in an entire genus [10]. A pangenome represents all the genes present across a species, a pan-genes can be subdivided into “core-genome” present in all the individuals in a species and “accessory genes” not present across all the isolates which is also called as “dispensable genome” [18]. For instance, three versions of *A. fumigatus* pangenome have been developed, emphasizing identification of azole drug resistance genes in *A. fumigatus* [19–21].

Therefore, a pangenomic approach is necessary to fully capture the genetic diversity present within the *A. flavus* species, particularly in agricultural environments, and can be used to perform in depth studies of a plethora of secondary metabolite gene clusters and their regulation. To accomplish this, present study was designed with three main objectives: (1) collection of large number of *A. flavus* isolates from diverse sources including maize plants and field soils; (2) whole genome sequencing of collected isolates and development of a pangenome; and (3) pangenome-wide association study (Pan-GWAS) to identify novel secondary metabolite cluster genes. This new *A. flavus* pangenome, here named as AflaPan, can serve as a new reference for comparative genomics studies by the Aspergillus research community with greater representation of the genetic diversity of this species than any single isolate reference can provide.

## Materials and methods

### Aspergillus Flavus isolates

**Fig. 1:**
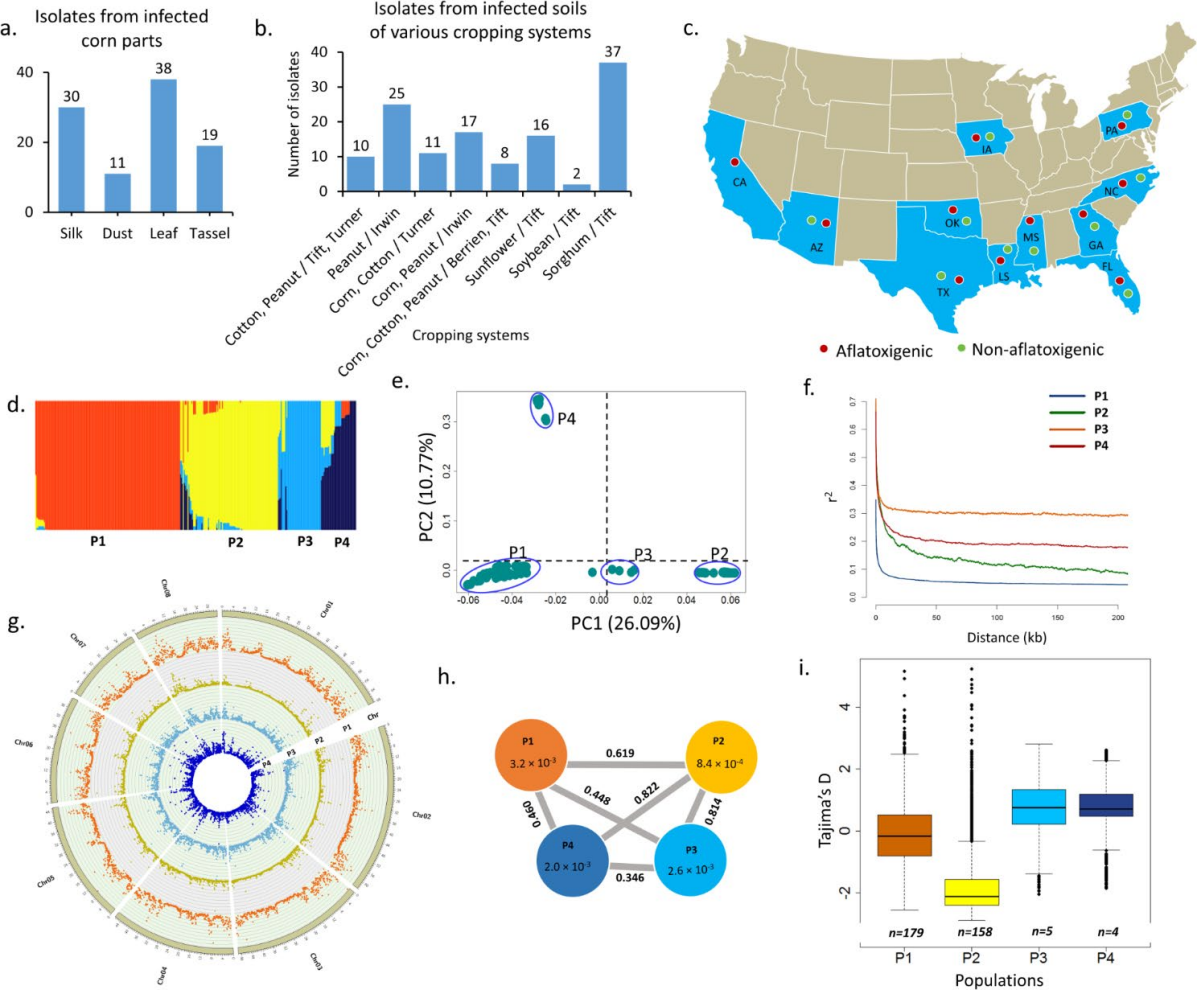
Isolate collection and diversity analysis. In this study, among 346 isolates, a total of 225 isolates were newly collected and sequenced, and 121 isolates were downloaded from NCBI-SRA. Of the 225 newly sequenced isolates, **a**) 98 isolates from various infected plant parts (Tassel, leaf, dust and silk) of corn, **b**) 127 isolates from soil samples from fields of various cropping systems of either rotation or continuous cropping of crops such as cotton, peanut, corn, sunflower, soybean, sorghum from three counties namely Tifton, Turner, Irwin, Berrien. **c**) A total of 346 isolates represents a total of 10 states of United States with representatives aflatoxigenic and non-aflatoxigenic isolates from each state. **d**) Population structure analysis identified four groups. Each color represents one group. Four groups are labelled as P1, P2, P3, and P4 respectively. **e**) Discriminant analysis of principle components confirmed four groups in the *Aspergillus flavus* population. **f**) Linkage disequilibrium (LD) decay estimates in each population. Pairwise linkage disequilibrium (r^2^) was calculated for each SNP in each population individually, in a window of 10Kb using plink. Four lines represents LD of four genetic populations. **g**) Circos plot illustrates nucleotide diversity (*π*) in each population. From outside to inside the tracks, chr represents 8 chromosomes of *A. flavus*, tracks from P1 to P4 represents nucleotide diversity present in four genetic populations of *A. flavus*. **h**) Means of nucleotide diversity (*π*) and *Fst* values calculated between the groups. The grey color links between the groups shows the *Fst* values between the groups. While the values inside the circles indicates the mean nucleotide diversity (*π*) present in that particular group. **e**) Box plots illustrates distribution of Tajima’D measures calculated in 10 kb window for the groups P1, P2, P3 and P4

A total of 225 isolates (98 from infected corns; 127 from soils) were newly collected and sequenced in this study. 98 isolates were associated with corn fields from infected plant parts of corn at harvest in Mississippi Delta, including 11 isolates from corn leaves, 38 from corn silks, 30 from corn tassels, and 19 from dust air-spora of corn combine harvester (Fig. 1a). Among the remaining 127 isolates from different field soils with diverse cropping systems in southern Georgia in the fall harvesting season, there were 10 isolated from corn and peanut rotation fields of Tift and Turner County, 26 continuous peanut fields in Irwin and Turner County, 11 from corn and cotton rotation fields of Turner County, 17 from corn and peanut rotation fields of Irwin County, 8 from corn, cotton and peanut rotation fields in Berrien and Tift County, 16 from continuous sunflower fields of Tift County, 2 from continuous soybean fields of Tift County, and 37 continuous sorghum field of Tift County (Fig. 1b).

For *A. flavus* isolation, protocol reported in [22] was followed with modification. Briefly, 10 g of soil sample was added to a bottle containing 99 mL of phosphate buffer solution. The bottles were placed on a shaker for 30 min, and afterwards, the mixtures were serially diluted with sterile phosphate buffer. In triplicate, 100 µL of each serial dilution was spread on 100 mm × 15 mm petri dishes containing Modified Dichloran Rose Bengal (MDRB) agar [23]. The inoculated plates were incubated in the dark for 3–5 days at 37 °C. After incubation, up to 20 *A. flavus* colonies for each sample were randomly selected from the MDRB plates and transferred to individual 60 mm × 15 mm Petri dishes containing potato dextrose agar amended with 0.3% β-cyclodextrin. The inoculated plates were incubated in the dark at 28 °C for 5–7 days and characterized for sclerotia and aflatoxin production.

In addition to 225 newly sequenced isolates, raw DNA sequence data for 121 isolates was also downloaded from previously reported genome assemblies from National Centre for Biotechnology Information Sequence Read Archive (NCBI-SRA). Finally, a total of 346 *A. flavus* isolates (225 new isolates and 121 from NCBI-SRA) representing 11 states of the United States were used for development of *A. flavus* pangenome (Fig. 1c). A detailed list of isolates with metadata, geographical site, mating types, and sclerotia types is provided in Supplementary Table 1.

### DNA extraction and whole genome sequencing

For short read sequencing for each isolate collection, a normal CTAB DNA isolation protocol was used [14]. Each isolate was cultured in yeast extract sucrose (YES) medium with 2% yeast extract, 1% sucrose for five days at 30 ° in the dark. Mycelial mats from each culture were collected and ground in a chilled mortar and pestle with liquid nitrogen. The ground mycelia (1–2 g) were then combined with 15 mL of CTAB extraction buffer (0.1 M Tris pH 8.0, 1.4 M NaCl, 20mM EDTA, 2% (w/v) CTAB, 4% (w/v) polyvinylpyrrolidone (PVP-40), and 0.5% (v/v) b-mercaptoethanol), mixed by inversion, and incubated in a water bath at 65 °C for 45 min with occasional inversion. The lysate was then combined with 15 mL of chloroform: isoamyl alcohol (24:1), mixed by inversion, and centrifuged at 8,000 · g for 15 min at 4 °. The upper phase was then transferred to a new 50 mL centrifuge tube. The chloroform separation was then performed a second time, and the upper phase was then combined with one volume of cold isopropanol for DNA precipitation. The DNA was then pelleted by centrifuging at 8,000 · g for 15 min at 4 °, and washed with 70% ethanol. The pellets were then dried and suspended in 100 mL TE buffer (10mM Tris pH 8.0, 1 mM EDTA pH 8.0). RNase-A was then added to a final concentration of 5 mg/mL and the samples were incubated at 37 ° for 1 h. The obtained DNA was then stored at -20 ° until used. Isolated DNA was quantified with both a Nanodrop ND-1000 spectrophotometer (ThermoFisher, Waltham, MA, USA) and a Qubit 3.0 fluorometer (ThermoFisher) and checked using gel electrophoresis.

Isolated DNA (500 ng) from each sample was then shipped to the Novogene Corp. (Sacamento, CA) for quality checking, sequencing, and initial data filtering [24]. Libraries (350 bp insert size) were generated using NEBNext® DNA Library Prep Kit (New England Biolabs, Ipswich, MA) following manufacturer’s instructions. In brief, initially dA-tailing followed by adapter, further the fragments (of 300–350 bp) were PCR enriched with P5 and indexed by P7 oligos. The quality of sequencing libraries was again checked on Qubit® 3.0 flurometer to determine the concentration of each library. Agilent® 2100 bioanalyzer was used to assess the insert size by using 1ng/ul DNA from sequencing libraries. Finally, the quantitative real time PCR (qRT-PCR) was performed to detect the effective concentration of each library. The libraries with appropriate insert size and with > 2mM concentration were qualified for high throughput sequencing on Illumina HiSeq 4000 platform. Pair-end sequencing data was generated for each isolate with the read length PE150 at each end. Sequencing data for these 225 isolates of *A. flavus* used in this study has been submitted to NCBI with bioproject ID: PRJNA915632.

### Genome assembly and annotations

The raw sequencing data quality analysis and trimming of adapter sequences for each isolate was carried out using Trimmomatic v0.40 [25]. Trimmed reads were assembled using SPAdes v.3.15.4 [26], with AF13 genome assembly as a reference using mismatch and short indel correction with the Burrow-Wheeler Aligner, BWA-MEM v.0.7.12 [27]. Assemblies were improved using Pilon v.1.22 [28]. The assembly summary statistics were calculated using QUAST v5.0.2 [29]. Functional annotation and gene prediction were performed using Funannotate pipeline v.1.8.14 [30]. In brief, the *A. flavus* assemblies were initially prepared for gene prediction and functional annotation. The small and repetitive contings (shorter than 500 bp) in the assembly were removed by using the function funannotate clean which implements minimap2 with “leave one out” methodology, where unaligned shortest contigs to N50 of assembly were filtered out with parameters percent coverage overlap (--cov = 95) and percent identity of overlap (--pident = 95). All assemblies were soft-masked using function funannotate mask with default method tantan RepeatMasker (v.4.0.8) for repetitive elements using Dfam and RepBase repeat libraries [31]. Masked assemblies were used for gene prediction using funannotate predict with the help of Evidence Modeler algorithm. Curated protein models from closely related species *Aspergillus oryzae* were provided as evidence for gene prediction.

### Species confirmation, identification of mating types, and types of sclerotia

Sclerotia are survival structures produced by *A. flavus* to survive in extreme environmental conditions [32]. Wild-type strains of *A. flavus* are known to produce sclerotia in culture after 5 days of incubation at ideal growth temperatures or a short period of cold storage. *A. flavus* can be categorized into three groups based on the type of sclerotia production: (1) no sclerotia, (2) small type (S) sclerotia (< 400 μm size, uniform, and rare to find), and (3) large type (L) sclerotia (> 400 μm. Large (L) type sclerotia are very common, not uniform, and different shapes. Notably, S- sclerotia producing *A. flavus* isolates are observed to be almost always positive for aflatoxin production, while L- sclerotia producers were observed to have little to no effect on the presence or concentration of aflatoxins [22].

Furthermore, S-type sclerotia strains, on average, produce more aflatoxin than the L-type sclerotia strains. In this study, to determine the sclerotia size of each *A. flavus* isolate, the samples were viewed under a microscope, and the microscope reticule was used to measure the size of the sclerotia. For sclerotia producing isolates, 100 sclerotia were measured to calculate average sclerotia size, and the average sclerotia size was used to assign the type of sclerotia production for *A. flavus*. We also confirmed the sclerotia types in individual isolates using the *cypA* and *norB* sequences. *A. flavus* and *A. parasiticus* are closely related species, but *A. flavus* produces only aflatoxins B1 and B2, while, *A. parasiticus* can produce B1, B2, G1 and G2. *A. flavus* genome is missing the portions of genes (*cypA* and *norB*) upstream to the polyketide synthase gene [33]. Therefore, we used *A. parasiticus* specific *cypA-norB* aflatoxin pathway gene cluster marker genes (NCBI accession no. AY371490) to detect the *A. parasiticus* isolates [34]. Based on blast results, a total of 225 isolates were confirmed as *A. flavus*, while a total of 39 isolates collected from soil were identified as *A. parasiticus* isolates. We collected a total of 346 *A. flavus* isolate genomes (225 from this study and 121 from NCBI-SRA) for construction of a pan-genome of *A. flavus*. We identified the large sclerotia (L) types using the *norB-cypA* region genomic sequence from strain NRRL3357 (NCBI accession no. AY566564), while, the short sclerotia (S) types were identified using locus of aflatoxin biosynthetic gene cluster from isolate AF70 (NCBI accession no. AY510453) [33].

In *Aspergilli*, *A. flavus* and *A. parasiticus* are heterothallic species with two mating type loci *MAT1-1* and *MAT1-2* located on chromosome 6 associated with sexual reproduction [35]. Mating types were identified among all isolates using the reported marker sequences. Mating types *MAT1-1* identified using complete cds sequence of mating-type protein MAT alpha 1 (NCBI accession no. EU357934), while the *MAT1-2* types were identified using the complete cds sequence of a putative DNA lyase gene also called as mating-type HMG (High Mobility Group)-box protein *MAT1-2* (NCBI accession no. EU357937) [36].

### Read alignment and variant calling

AF13 reference genome (v1.0, Gene Bank assembly accession number GCA_014117485.1) was used for mapping and variant calling. The genome files for AF13 reference genome were downloaded from NCBI from bio-project ID PRJNA606266 [14]. Sequence reads for 346 *A. flavus* isolate were aligned to AF13 reference genome using Burrows-Wheeler Aligner (BWA v.0.7.17) [27]. The alignment files were converted into BAM files using Samtools v.1.10 [37]. Picard tools v.2.18.3 (http://broadinstitute.github.io/picard) used to mark the PCR duplicates using MarkDuplicate. HaplotypeCaller implemented in GATK (v.4.1.0) was used to call the SNPs using AF13 as a reference genome [38]. VariantFiltration followed by SelectVariants workflow was used to filter variants using parameters [-window-size = 10, -QualBy-Dept < 2.0, -MapQual < 40.0, -Qscore < 100, -MapQualityRankSum <-12.5, -StrandOddsRatio > 3.0, -FisherStrandBias > 60.0, -ReadPosRankSum <-8.0.].

### Whole-genome phylogeny estimation

SNV (Single Nucleotide Variants) based phylogenetic tree was constructed by filtering the variants at minor allele frequency 0.25 and the loci with zero coverage in any isolate. Variants from each isolate were concatenated and used as input in RAxML v.8.2.12 to construct maximum likelihood (ML) tree [39]. Annotations for origin, source, toxicity, sclerotia types, mating types, genome size (Mb), and number of scaffolds for each isolate were plotted on phylogenetic tree using iTOL (Interactive Tree of Life) [40]. The final tree was visualized in iTOL by customizing the parameters. Genetic clusters among 346 *A. flavus* isolates identified using discriminant analysis of principle components [41]. The PCA plot was visualized using ggplot2 in R version 4.2.2 [42].

### Population structure and diversity analysis

Population structure of 346 *A. flavus* isolates was studied by identifying optimum number of groups (*k*) corresponding to the lowest Bayesian information criterion (BIC). The STRUCTURE v.2.3.4 software was used to study the population structure and admixture [43]. Diversity parameters such as nucleotide diversity (*π*), fixation index (*F*_*ST*_), and Tajima’s D diversity index [44] were analyzed for each genetic cluster to understand genetic diversity and distinctness of genetic clusters. VCFtools v.0.1.13 was used to calculate the genetic diversity parameters (nucleotide diversity (*π*), fixation index (*F*_*ST*_), and Tajima’s D) in a non-overlapping window of 10,000 bp [45]. Nucleotide diversity (*π*) is used as measure of variability of SNPs nucleotides within individual genetic clusters. Nucleotide diversity (*π*) calculated in VCFtools using parameters --site-pi --window-pi 10,000. Tajima’s D diversity index allows us to see whether a population is neutrally evolving or whether it is under selection. Tajima’s D was calculated in VCFtools with parameters --TajimaD 10,000. Genome-wide fixation index (*Fst*) is a measure of population differentiation. Fst ranges from 0 to 1, where is *Fst* = 0 means two populations are genetically similar. While, Fst = 1 means the populations are significantly genetically dissimilar to each other. Fst was calculated using VCFtools with parameters --weir-fst-pop --fst-window-size 10,000. All diversity statistics was visualized using ggplot2 in R. The linkage disequilibrium decay for each population was calculated using VCFtools in 10000Kb window. The linkage disequilibrium (*r*^*2*^) was plotted against the distance between the two loci in the equilibrium (Kb).

### Development of a pangenome, AflaPan, and annotation of orthogroups

The protein sequences from 346 isolates obtained from funannotate were used to identify the orthologous gene clusters using OrthoFinder [46]. Protein sequences from the AF13 reference genome were also included to identify the functions of orthologous gene clusters in AflaPan. If a gene sequence from AF13 genome is grouped in an orthologous cluster then the whole cluster is annotated as the annotation of the grouped sequence from AF13 reference genome. However, some orthologous gene clusters were not grouped with single sequence of AF13 genome. We called these orthologous gene clusters as a non-reference gene clusters when compared with AF13 reference genome. A customized bash script was used to extract the most extended protein sequence for every orthologous group to functionally annotate the orthogroups. Blast + was used to find the highest similar proteins against SwissProt (https://www.expasy.org/) [47] and FunigDB (https://fungidb.org/) [48]. The sequences remain functionally annotated with annotations from AF13 reference genome and FungiDB were further annotated using InterProScan (https://github.com/ebi-pf-team/interproscan) [49]. We used the criteria E-value cutoff of 1 × 10^5^, percent identity > 70%, minimum query coverage > 50% and minimum subject coverage > 50% [19]. The novel orthogroups were included in the pangenome only if they are present in at least 5% of the isolates in the study, to avoid false gene prediction. The presence/absence matrix of orthologous groups used to describe the framework of AflaPan genome. Further the presence/absence matrix was used for Pan-GWAS analysis, to check the utility of the AflaPan genome.

### Estimation of aflatoxin content (ppb)

Up to 20 *A. flavus* colonies for each isolate were randomly selected from the Modified Dichloran Rose Bengal (MDRB) plates and transferred to individual 60 mm × 15 mm petri dishes containing potato dextrose agar amended with 0.3% β-cyclodextrin. The inoculated plates were incubated in the dark at 28 °C for 7 days. After incubation, evaluated for aflatoxigenicity using three cultural methods using yellow pigment test, UV fluorescence test, and ammonia vapor test [22]. The percentage of aflatoxin-producing colonies for each sample was calculated based on the qualitative analysis of the three cultural methods.

To quantitate the aflatoxin production by *A. flavus* isolates, aflatoxins were extracted from agar plugs of 7-day old cultures and analyzed by Enzyme Linked Immuno-Sorbent Assay (ELISA). First, agar plugs of fresh cultures were extracted at 1:5 ratio with methanol-water (70:30, *v*/*v*). The extracts were filtered through 0.25 μm pore-size nylon syringe filters. The filtrates were stored at 4 °C until use. For ELISA, Veratox® for Aflatoxin kits (Neogen Inc., Lansing, MI, USA) used to determine the total aflatoxin concentration. If the results exceeded the quantitative parameters of the protocol, the filtrates were serially diluted and re-analyzed. The final aflatoxin content (ppb) was calculated using the dilution factor determined by number dilutions. A Neogen Stat-Fax 303 Plus microwell reader was used to measure the absorbance of the ELISA reaction and to calculate the final concentration of aflatoxins in solution [22].

### Pan-GWAS analysis to identify the aflatoxin production associated gene clusters

The presence/absence variance matrix (PAV) from 17,855 orthologous gene clusters of AflaPan and phenotyping data generated for aflatoxin production was used for pan-genome wide association analysis. The presence/absence matrix of 1/0, where ‘0’ represents absence of orthogroups, and ‘1’ means presence of orthogroups in a particular isolate. The objective behind Pan-GWAS analysis was to identify the association of accessory genes with aflatoxin production in *A. flavus*. Pan-GWAS analysis was perform using mixed-linear-model (MLM) implemented in TASSEL v.5.0 [50]. MLM calculates both fixed and random effects in the population. For MLM, a kinship (K) was calculated using PAV matrix and used jointly with population structure (Q). The Q + K approach helps to increase the power of genome-wide association analysis [51]. A Bonferroni threshold was calculated at *P*-value 0.05, Bonferroni threshold = 0.05/17,855 (Orthogroups in AflaPan) = 2.8 × 10^− 6^. The orthogroups showing P value less than or equal to Bonferroni threshold of 2.8 × 10^− 6^ were called as significantly associated orthogroups [52]. GWAS results were visualized using Manhattan plots and Quintile-Quintile (QQ) plots generated in the R package ‘CMplot’ [53].

### Availability of data and materials

Sequencing statistics, assembly statistics, SNP statistics, and Pan-GWAS analysis are provided in the attached supplementary files. The newly developed genome assemblies for 225 isolates used in this study are available at https://zenodo.org/deposit/7615243. Raw sequencing data and metadata for each isolate is available through National Center for Biotechnology Information (NCBI) - Sequence Read Archive (SRA) with Bioproject ID PRJNA915632. Newly sequenced 225 fungal isolate cultures are available upon request by contacting the corresponding author. The 121 isolates from public data can be requested from corresponding authors of respective articles [11, 24, 54].

## Results

### De novo genome assemblies of *A. Flavus* isolates

A total of 225 isolates were newly collected and sequenced as part of this study (Fig. 1a and b). In addition, we downloaded raw sequencing data for 121 previously sequenced isolates from NCBI sequence read archive. Among these, 7 isolates were sequenced by Fountain et al. (2020b) [24], 94 sequenced for studying pan-metabolomics [11], and 20 draft genomes from [54]. Overall, a total of 346 isolate genomes were included in this study, representing the diversity from 11 states (California, Arizona, Oklahoma, Texas, Louisiana, Mississippi, Georgia, Florida, North Carolina, Pennsylvania and Iowa) of the United States and soils of crops including peanut, sunflower, soybean, sorghum, corn, sesame and cotton. Of these 346 isolates, 177 were aflatoxigenic and 169 were non-aflatoxigenic. For sclerotia, there were 128 isolates with large sclerotia (L-strains) and 218 isolates showing small sclerotia (S-strains). Finally, for mating types, 159 showed mating type *MAT1-1* and 187 isolates showed mating type *MAT1-2* (Supplementary Table 1).

De novo genome assemblies were developed for 346 *A. flavus* isolates using paired end Illumina sequencing reads. The mean number of contigs and scaffolds in the assemblies for soil isolates were 597 and 570, with average of 726 and 697 contigs and scaffolds, respectively. Average length of contigs and scaffolds of soil isolates was 89 kb and 98 kb, respectively. The assemblies of isolates collected from corn dust had a total of 111 and 131, contigs and scaffolds, respectively, the lowest number of the newly sequenced isolate genomes. In contrast, the assemblies of isolates collected from corn leaf tissues had the highest number 151 and 167 of contigs and scaffolds, respectively. The assembly mean contig N50 and mean scaffold N50 were 403,194 bp and 523,939 bp. Mean genome size of the assemblies of corn isolates and soil isolates was 36.9 Mb and 37.6 Mb, respectively. It is observed that soil genomes are slightly longer than corn isolates, with maximum 43.2 Mb genome size in soil isolates and maximum 38.5 Mb in corn isolates. Among the corn isolates there was no significant difference in number of contings and scaffolds collected from different corn parts such as silk, leaf, tassel and dust, and no significant difference in the genome sizes of corn isolates was observed (Table 1; Supplementary Table 1).

**Table 1:** Summary of newly sequenced genome assemblies from corn and soil samples used for development of an *Aspergillus flavus* pangenome (AflaPan)

Feature	Soil isolates			Corn isolates		
	Minimum	Maximum	Average	Minimum	Maximum	Average
Scaffolds	149	7,949	696.8	111.0	1230	269.7
Contigs	170	8,104	726.3	146.0	1,436.0	295.7
Genome size (Mb)	36.6	43.2	37.6	36.5	38.5	37.0
Average scaffold size (Kb)	5.4	248.6	98.0	27.8	274.1	161.5
Average contig size (Kb)	5.3	217.9	87.8	26.8	253.1	144.4
Contig N50 (Kb)	11.1	1,439.6	307.4	346.3	1,054.5	603.7
Scaffold N50 (Kb)	11.1	2,316.2	406.2	378.6	1,367.5	775.6

To perform population genomics studies, we retrieved ~1.02 million single nucleotide variants (SNVs) using AF13 as reference, approximately 28 SNVs per kilo bases (kb) among 346 *A. flavus* isolates. Large number of SNVs across the isolates indicated prominent genetic diversity in the species of *A. flavus*. On an average of 1,021,529 SNVs per isolate (ranged from 795,241- 1,027,945 SNVs) were used for diversity analysis. An average of 4,871 SNVs showed heterozygous calls, and 1,016,658 SNVs were homozygous per isolate. Only homozygous SNV calls were used for population genomics studies (Supplementary Fig. 1; Supplementary Table 1).

### Diversity in the population of 346 *A. flavus* isolates

A total of four genetic clusters (*K* = 4) were identified in the collection of 346 *A. flavus* isolates using population structure analysis. The largest genetic cluster (P1) included 179 isolates, with majority of isolates from soil. Second largest cluster (P2) included 158 isolates grouped from soil as well as corn. Third and fourth clusters were minor with merely 5 and 4 isolates, respectively. Highly toxigenic isolates collected from the state of Louisiana were grouped in clusters 3 and 4 (Fig. 1d). Four principle components identified using discriminant analysis of principle components further confirmed the presence of four genetic clusters (Fig. 1e). Linkage-disequilibrium (LD) decay for each genetic cluster was calculated and plotted *r*^*2*^ values against distance between two SNVs (Kb) using ggplot2. Rapid LD decay was observed for genetic cluster P1 (10 Kb), followed by genetic cluster P3 (12 Kb), cluster P4 (18 Kb) and cluster P2 (25 Kb). The population growth results in reduction of LD the largest genetic cluster (P1) showed least LD decay (Fig. 1f).

Genome-wide nucleotide diversity (*π*), fixation index (*F*_*ST*_) and Tajima’s D diversity was analyzed individually for each cluster to understand the distinctness of genetic clusters. Genome-wide nucleotide diversity (*π*) for each cluster was visualized using circos plot, showed significant number of hot spots with high nucleotide diversity in each cluster (Fig. 1g). Highest average nucleotide diversity (*π* = 3.2 × 10^− 3^) was observed for cluster P1, followed by cluster P4 (*π* = 2.6 × 10^− 3^), and cluster P3 (*π* = 2.0 × 10^− 3^). The lowest nucleotide diversity was observed in genetic cluster P2 (*π* = 8.4 × 10^− 4^) (Fig. 1h). Further, the chromosome wise nucleotide diversity across 346 isolates was represented using boxplots. Chromosome 8 showed highest nucleotide diversity (mean *π* = 5.0 × 10^− 3^, with range 1.9 × 10^− 2^ – 3.8 × 10^− 7^), followed by chromosomes 5, 6 and 7 (mean *π* = 3.7 × 10^− 3^, with range 1.85 × 10^− 2^ – 5.5 × 10^− 7^), chromosomes 3 and 4 (mean *π* = 3.5 × 10^− 3^, with range 1.7 × 10^− 2^ – 5.4 × 10^− 7^), and chromosomes 1 and 2 (mean *π* = 3.2 × 10^− 3^, with range 1.9 × 10^− 2^ – 2.5 × 10^− 5^) (Supplementary Fig. 2). The population differentiation was investigated by calculating fixation index (*F*_*ST*_) between the pairs of genetic clusters. Fixation index (*F*_*ST*_) value can range from 0 to 1. Where, *F*_*ST*_ =0 indicates the two clusters are completely sharing each other, whereas *F*_*ST*_ =1 means there is no sharing or complete differentiation between two clusters. In this study, higher *F*_*ST*_ values (> 0.6) of the cluster P2 with clusters P1, P3 and P4 indicated that P2 cluster is most differentiated cluster than other three clusters. For instance, fixation index (*F*_*ST*_) of 0.822 was recorded between clusters P2 and P4, followed by 0.814 between P2 and P3, and 0.619 between P2 and P1. Lowest fixation index (*F*_*ST*_ =0.346) was recorded between the clusters P3 and P4, followed by P1 and P3 (*F*_*ST*_ =0.346), P1 and P4 (*F*_*ST*_ =0.460) (Fig. 1h).

Genome-wide Tajima’s D diversity index was calculated for each cluster to study the abundance of rare alleles in each cluster. Tajima’s D diversity helps to determine whether the cluster is evolving neutrally, or under selection pressure. Tajima’s D = 0, means the cluster is evolving neutrally without any evidence of selection, Tajima’s D < 0, means excess of rare alleles in the clusters or population expansion, Tajima’s D > 0, means scarcity of rare alleles in cluster or population contraction. Tajima’s D for cluster P1 was − 0.13, indicating that cluster P1 is evolving neutrally with partial selection sweep. Interestingly, Tajima’s D for cluster P2 was − 1.77, indicated abundance of rare alleles in P2. The Tajima’s D for cluster P3 (0.81) and P4 (0.81) was greater than zero indicating that these two populations lack rare alleles and therefore the clusters are resulted in the population contraction (Fig. 1i). Chromosome wise Tajima’s D index was higher for chromosome 6 and 8 (Supplementary Fig. 3).

### Whole-genome phylogeny and characteristics of genetic clusters

**Fig. 2:**
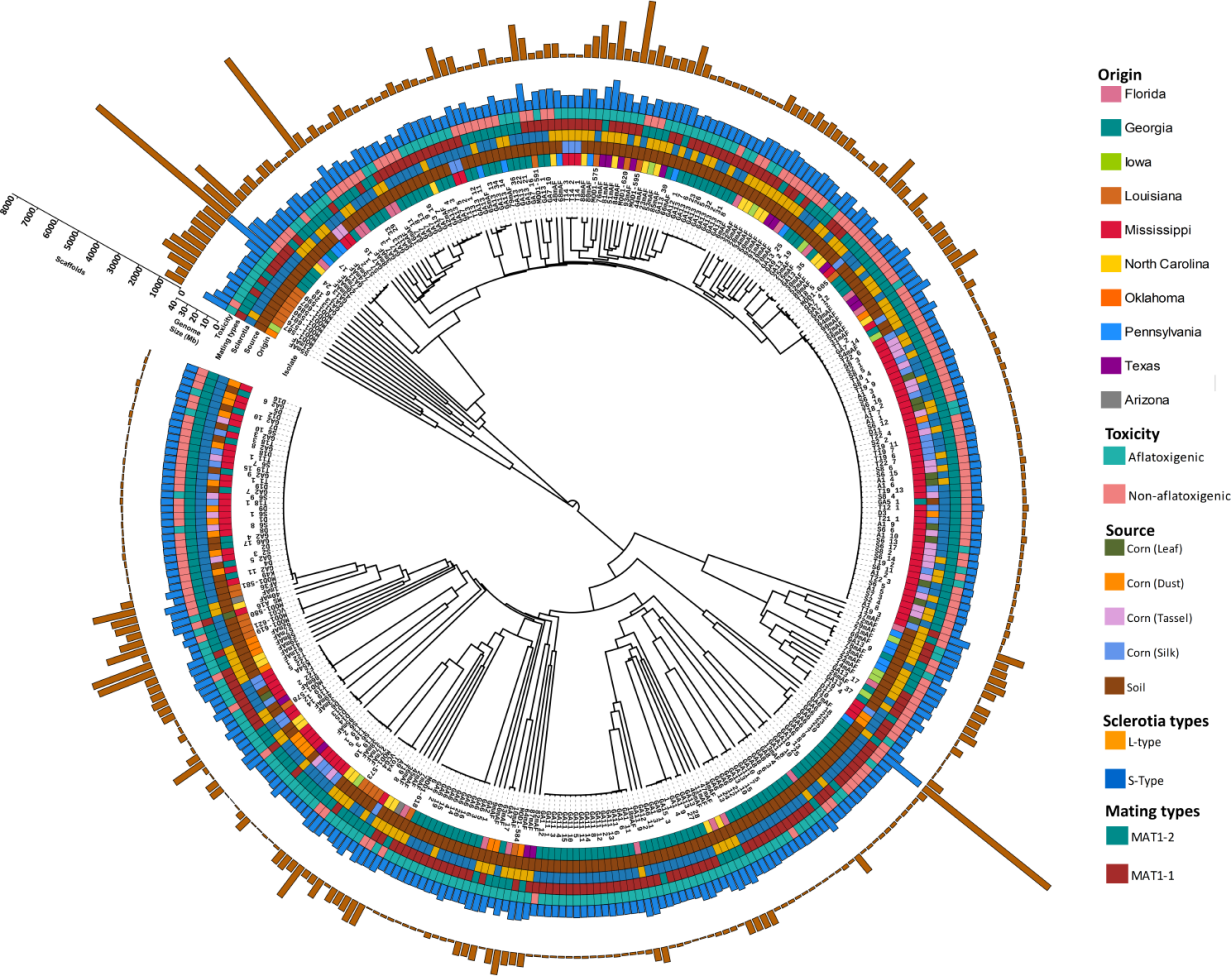
Whole genome phylogeny of soil and corn *A. flavus* isolates. A whole genome phylogeny constructed using ~1.02 million SNPs called on whole genome sequencing data on 346 isolates. The tracks from inside to outside shows information of isolates on name of isolate, origin, source, sclerotia types, mating types, toxicity, genome size and number scaffolds in each genome. (1) Isolates- represents the name of each isolates, (2) Origin- illustrates the geographic site of each isolate from 10 states of United States, (3) Source- shows the information on whether it is isolated from corn (tassel, leaf, silk, dust) and soil, (4) Sclerotia- shows information on sclerotia types short sclerotia (S-types) and long sclerotia (L-types), (5) Mating types- explains the diversity of isolates based on two mating types namely, *MAT1-1* and *MAT1-2*, (6) Toxicity of isolates- illustrates the diversity based on aflatoxigenic (toxin producers) and non-aflatoxigenic (not produce toxins), (7) Genome size (Mb)- the bar plots shows the genome size of each isolate in million base pairs (Mb), (8) Scaffolds- number of scaffolds in genome assembly of each isolate

Whole genome phylogeny was constructed and confirmed the presence of 4 clusters within population of 346 *A. flavus* isolates. Morphological characteristics of each cluster were investigated for their origin (geographical site), source (soil or corn), mating types, sclerotia types, toxigenicity, genome size (Mb), and number scaffolds in each assembly (Fig. 2). In the major cluster P1, the majority of isolates were from soil (136) and 43 were from corn. In cluster P1, a total of 126 isolates had small sclerotia (S-types), and 53 had large sclerotia types. In case of mating types, cluster P1 included 94 isolates with mating type *MAT1-1*, whereas 85 were *MAT1-2*. Interestingly, in cluster P1, a total of 111 isolates were aflatoxigenic and 68 were non-aflatoxigenic. Second major cluster P2 included a total of 103 isolates from soil and 55 from corn. In this cluster 58 isolates were aflatoxigenic and 100 isolates were non-aflatoxigenic. There were 74 isolates with large sclerotia (L) types and 84 with small sclerotia (S) types. In case of mating types, 61 isolates showed (*MAT1-1*) types and 97 showed (*MAT1-2*) types. In cluster P1, majority of isolates were aflatoxigenic, whereas in P2 majority of isolates were non-aflatoxigenic. Cluster P3 and P4 were minor genetic clusters with 5 and 4 isolates, respectively. In genetic cluster P3 all isolates were from collected from soil with aflatoxigenic, small sclerotia types, and four with *MAT1-1* and one *MAT1-2* mating types. In cluster P4, three isolates were aflatoxigenic and a one isolate was non-aflatoxigenic from the state of Iowa. All aflatoxigenic isolates in cluster P3 and P4 were from the states of Louisiana and Oklahoma (Fig. 2).

### 310 kb insertion is rare and identified only in soil samples

Recently a highly toxigenic *A. flavus* strain AF13 was sequenced, and a 310 kb insertion was identified in AF13 genome, but was absent in isolate NRRL3357. This 310 kb insertion includes a bZIP transcription factor named *atfC* [14].

The presence of 310 kb region was analyzed across 346 isolates, there were only 5 isolates with this 310 kb insertion. Interestingly, all these 5 isolates are collected from soil samples in the state of Louisiana, and all are toxigenic isolates grouped in cluster P3 and P4 (Fig. 2). However, there were about 57% shared identity with this 310 kb insertion among the other 341 isolates. Therefore, 310 kb insertion is rare in *A. flavus* species and the isolates with this insertion are highly toxigenic [14] (Supplementary Table 1).

### AflaPan contains 7,315 core and 10,540 accessory genes

**Fig. 3:**
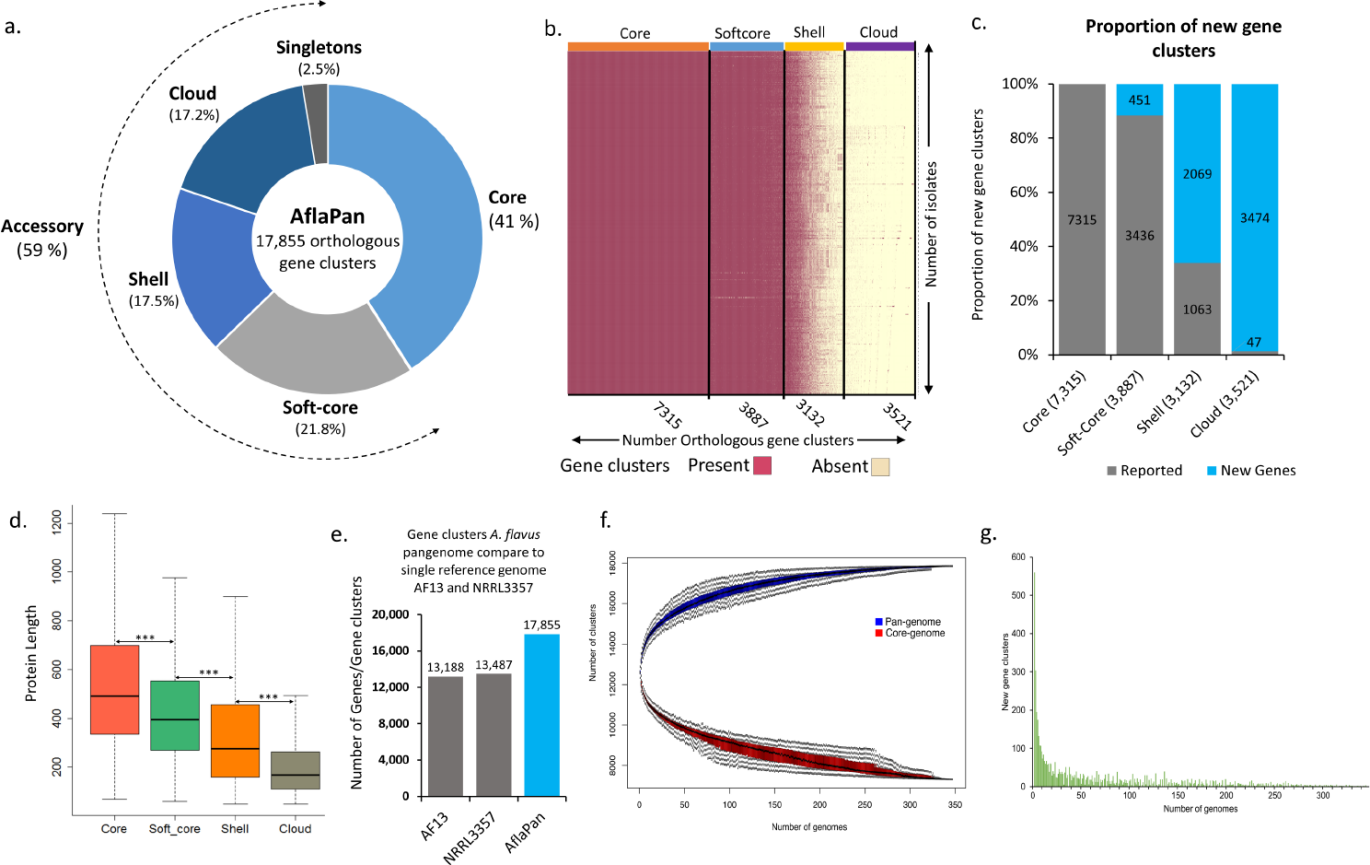
Overview of the pangenome of *Aspergillus flavus* (AflaPan). **a**) Total orthologous gene clusters and proportions of core, soft-core, shell, clouds and singletons genes in AflaPan genome. **b**) A bi-color plot illustrates presence/absence matrix of 17,855 orthologous gene clusters identified across 346 *A. flavus* genomes. Vertical axis represents number of genomes, while horizontal axis represents number of orthologous gene clusters, categorized into core, soft-core, shell and cloud. Where, core orthologous gene clusters are present in all 346 genomes, softcore (orthogroups present in > 95% of the isolates), shell (orthogroups present in 5–95% of the isolates) and cloud (orthogroups present in less than 5% of the isolates) genomes. **c**) Stacked bar graph shows proportion of non-reference gene clusters when compared with current reference genome AF13. **d**) Comparison of gene modules of AflaPan genome and current reference genomes of *A. flavus*, AF13 and NRRL3357. In AF13 and NRRL3357 there are a total 13,188 and 13,487 genes annotated respectively, in AflaPan we annotated 17,855 orthogroups, **e**) Comparison of length of protein sequences in core and accessory genomes. There is significant difference between the length of protein sequences in core and accessory genomes. The length of protein sequences of core genome is significantly longer than accessory genome. **f**) Orthologous gene clusters in accessory and core genome increases with increasing number of genomes. Blue and red color represents pan- accessory and core genes respectively. The black dotted line represents the range. **g**) New gene clusters were decreased with increasing the number of genomes in AflaPan genome. After adding 300 genomes the there was no significant increase in the new genes in AflaPan

Genome assemblies of 346 isolates were used to develop pan-genome framework for *A. flavus*, called AflaPan genome. A pan-genome represents total genes in the species, including core genes shared by all the individuals of the species and accessory genes that are not present in all the individuals of the species. In this study, we collected 346 genomes of *A. flavus* and identified 17,855 unique orthologous gene clusters in this pangenome, called AflaPan genome. In AflaPan, of the 17,855 orthologous gene clusters, there were 7,315 orthologous gene clusters as core genome (41.1% of the pangenome) present in all 346 genomes, 3,887 as softcore orthogroups in > 95% genomes (21.7% of the pangenome), 3,132 as shell genes in 5–95% genomes (17.5% of the pangenome), and 3,521 as cloud genes present in less than 5% of the genomes (19.7% of the pangenome) (Fig. 3a and b). We also report here 5,994 non-reference gene clusters that were not annotated in the AF13 reference genome [14]. Of the new or non-reference gene clusters, 451 were identified in soft-core (12% of total soft-core genes), 2,069 (66% of total shell genes) in shell and 3,474 (99% of total cloud genes) in cloud (Fig. 3c).

The gene clusters in the AflaPan genome were compared with the gene models in current reference genomes AF13 and NRRL3357 of *A. flavus*. Reference genomes AF13 and NRRL3357 contains of 13,188 and 13,487 annotated gene models, respectively. However, in AflaPan, a total of 17,855 unique orthologous gene clusters were annotated. The AflaPan genome includes more diverse genome segments that were missing in current single reference genome assemblies (Fig. 3d).

Significant differences between the lengths of protein sequences were observed in core and accessory genomes. The average protein length in core genome was 579 amino acids (AAs), with a range of 68 to 6885 AAs. While, the average protein lengths in soft-core genome was 463 AAs, with range of 60 to 7781 AAs. Average protein length in shell genome was 361 AAs, with the range of 50 to 3917 AAs. In clouds and singletons, the average protein length was 228 AAs and 233 AAs, respectively. Therefore, the conservative proteins were longer in length. Overall, the average protein length was decreased from core-softcore-shell-clouds and singletons (Fig. 3e; Supplementary Table 4; Supplementary Table 5).

Furthermore, the Aflapan was closed and captured comprehensive genetic diversity of the intraspecies of *A. flavus*. Any addition of *A. flavus* genomes will not substantially increase the number of pan-genes or core genes of *A. flavus* pan-genome (Fig. 3f). The number of pan-genes and core genes will be not substantially increased beyond this large collection of 346 *A. flavus* genome in this AflaPan (Fig. 3g).

### Toxin producing gene clusters in AflaPan

The orthologous gene clusters in AflaPan were annotated using blasts in InterProScan, SWissProt, and FungiDB databases. A total of 17,038 orthogroups were annotated using FungiDB database. There were 12,691 orthologous gene clusters annotated using SwissProt and 15,740 annotated using InterProScan. Therefore, there were a total of 12,500 orthogroups annotated using all three databases and 399 orthologous gene clusters were not annotated using either of the three databases (Supplementary Table 5). Based on the annotations a total of 89 toxins producing orthologous gene clusters were identified in core genome. Toxins producing gene clusters (TPCs) in core genome include 3-hydroxyacyl-CoA dehydrogenase-like protein, 6-hydroxy-D-nicotine oxidase, acyl-CoA dehydrogenase, satratoxin biosynthesis SC3 cluster protein, astacin-like metalloprotease toxin, aflatoxin B1 (AFB1) aldehyde reductase, viriditoxin biosynthesis cluster protein D (*VdtD*), latrotoxin producing gene cluster (*LTX*), AM-toxin biosynthesis protein or cytochrome P450 monooxygenase AMT3, AAL-toxin biosynthesis cluster protein, cercosporin toxin biosynthesis cluster, viridicatumtoxin synthesis proteins (*vrtL* and *vrtT*), aflatoxin biosynthesis protein O, and transcription activator AMTR1 (AM toxin). Cytochrome P450 monooxygenase is potent mycotoxins producing gene clusters, and in AflaPan, we identified *ALT8*, *aflU*, *aflV*, and *AKT7* toxin producing cytochrome P450 monooxygenases. TPCs in core genome indicated that these clusters are widely present in the species *A. flavus* and annotated in reference genomes AF13 and NRRL3357.

A total of 43 TPCs identified in soft-core genome including 5 new gene clusters integrated from soil isolates of Louisiana and Oklahoma. New TPCs in soft-core genome included toxin subunit YenA2, taurine hydroxylase-like protein SAT17, and cytochrome P450 monooxygenase AKT7. Similarly, in shell genome, a total of 45 TPCs were annotated. Interestingly, a total of 23 new gene clusters were identified in shell genome linked with toxin production. New TPCs in shell genome include MFS gliotoxin efflux transporter (*gliA*), called gliotoxin biosynthesis protein A, alpha-latrocrustotoxin-Lt1a, killer toxin subunits alpha/beta, double-stranded DNA deaminase toxin A, and acyl-CoA dehydrogenase AFT10-1. Particularly, in shell genome there were 33 new TPCs integrated from soil isolates and 3 were from corn samples. In cloud genome, a total of 36 TPCs were identified, and all were new or non-AF13 TPCs. Among singletons we identified 8 new TPCs and all were originated from soil samples of Georgia and Mississippi. The TPCs in singletons included C3 and PZP-like alpha-2-macroglobulin domain-containing protein, ADP-ribosylating toxin CARDS (Community-Acquired Respiratory Distress Syndrome toxin), norsolorinic acid reductase (*aflE*), FAD-dependent monooxygenase, aflatoxin cluster transcriptional coactivator (*aflS*), cytochrome P450 monooxygenase (*alfG*), and abhydrolase domain-containing protein AFT2. Here we observed that the extended genome portions of soil included new toxin producing genes (Supplementary Table 5). In order to check the association of new orthologous gene clusters with toxin production and secondary metabolite production, AflaPan-genome wide association analysis (AflaPan-GWAS) was conducted using phenotypes of total aflatoxins produced.

### Secondary metabolite producing gene clusters

*A. flavus* also produces a variety of secondary metabolites which may be useful for pharmaceutical industries. In current AF13 and NRRL3357 reference genomes of *A. flavus* [14] there were 80 and 78 secondary metabolite producing gene clusters. In this AflaPan genome, there were a total of 160 secondary metabolite producing gene clusters annotated for 10 important secondary metabolites, including aflatrem (14), aflavarin (3), aflatoxin (63), asparasone (6), cyclopiazonic acid (25), ditryptophenaline (3), kojic acid (3), leporins (4), piperazines (19), and ustiloxin (20).

Aflatrem is tremorgenic mycotoxin, the only class of mycotoxins impacting central nervous system [55]. In AflaPan, a total of 14 orthogroups were annotated as aflatrem biosynthetic gene clusters. Among them, 8 orthogroups identified in core, and 5 in soft-core and 1 in shell genome. It indicated that aflatrem producing gene clusters are highly conserved in *A. flavus*.

Aflavarin is also an important sclerotial metabolite exhibiting insecticidal activity [55]. In AflaPan, a total of 3 aflavarin producing orthologous gene clusters were annotated, and there were two in core genome and one in cloud genome.

Aflatoxin is a potent mycotoxin produced by *A. flavus* classified as polyketide synthase (PKS) backbone genes. Based on annotation, there were a total of 63 aflatoxin producing secondary metabolite gene clusters identified in this AflaPan genome. Among them, 20 were identified in core, 10 in soft-core, 21 in shell genome, 9 in cloud, and 3 as singletons. Aflatoxin producing gene clusters are uniformly distributed in core and accessory genomes of AflaPan. Moreover, Pan-GWAS analysis was conducted using phenotyping data of aflatoxin produced by 225 isolates, which uncovered 391 orthogroups associated with toxin production, including these 63 aflatoxin producing secondary metabolite gene clusters.

Asparasone is another sclerotial metabolite of structural class PKS [56]. In AflaPan, a total of 6 asparasone producing orthologous gene clusters were identified. Among them, there were one in each core and shell, 2 in soft core, and 2 in cloud genome of AflaPan. Cyclopiazonic acid (CPA) is an indole-tetramic acid mycotoxin, which belongs to structural group dimethyl tryptophan synthases (DMATs) or hybrid PKS (polyketide synthases)-NRPSs (non-ribosomal peptide synthetases) produced by a number of species of *Aspergillus* [56]. A total of 25 CPA producing orthogroups were identified in AflaPan. Among them, 7 identified in core, 4 in soft core, 6 in shell, and 8 in cloud genomes of AflaPan. Ditriptophenaline (DTP) produces diketopiperazine alkaloid in *A. flavus*. There were three DTP producing orthogroups identified in cloud genome of AflaPan.

Kojic acid (5-hydroxy-2-hydroxymethyl-1, 4-pyrone) belongs to structural group oxydoreductases, a skin lightning agent widely used in cosmetics industries. Moreover, it also has antibacterial activity and therefore used as pharmaceuticals, pesticides and insecticides [57]. In AflaPan, three orthologous gene clusters were identified associated with Kojic acid biosynthesis. Two of them were in core and one was identified in cloud genome of AflaPan. Leporin A is an antiinsectan N-methoxy-2-pyridone sclerotial metabolite isolated from the sclerotia of *A. leporis*. Leporin belongs to structural group hybrid PKS-NRPS. In AflaPan, four orthogroups were identified linked with leporin biosynthesis. Among them, three were in soft-core genome and one was identified as a singleton in soil isolate GA6_18.

Piperazines are class of saturated N-heterocycles, acting as central nervous system stimulants widely used in medicinal chemistry [58]. In AflaPan, a total of 19 orthogroups identified as piperazines biosynthesis gene clusters. Among them, there were 5 identified in core, 2 in soft-core, 2 in shell, 9 in clouds, and one as singleton identified in soil isolate GA6_18. Interestingly, in AflaPan, there were 10 new orthogroups identified associated with Piperazines biosynthesis. Ustiloxin B is a mycotoxin with cyclic peptide belonging to structural group ribosomally synthesized and post-translationally modified peptides (RiPPs). In AflaPan, there were 20 orthogroups annotated as Ustiloxin B biosynthetic clusters. Among them, there were 9 identified in core genome, 5 in soft-core genome, 3 in shell genome, and 3 in cloud genome of AflaPan (Supplementary Table 6).

### Pan-GWAS uncovered gene clusters associated with aflatoxin biosynthesis pathways

**Fig. 4:**
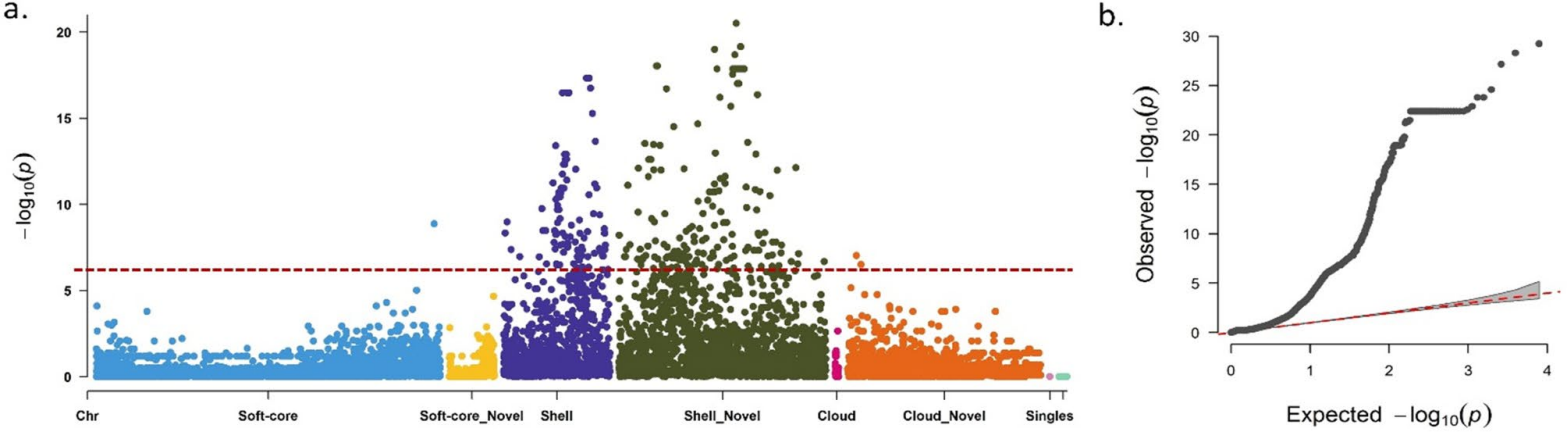
Pan-GWAS analysis for isolate aflatoxin production in culture. **a**) Manhattan plot for aflatoxins (ppb), **b**) QQ plot for total toxins (ppb). On X-axis represents the portions of soft-core, shell, clouds and singletons. Y-axis represents the *p* values transformed as –log10(*p*). The non-reference genes when compared with AF13 reference genome in the accessory genomes were visualized in separate groups with suffix ‘non-reference’. For instance, soft-core non-reference represents the new genes in soft-core genome, Shell non-reference represents new genes in shell genome, Cloud non-reference represents new genes in cloud genome. Single non-reference represents non-reference singletons. The Bonferroni threshold is marked at –log10(*p*) = 2.8 × 10^− 6^ with dotted red line

Pan-GWAS analysis using PAV matrix of 17,855 orthogroups and the newly collected 225 isolates of *A. flavus* uncovered new or non-reference and existing genes associated with aflatoxin production. Pan-GWAS analysis identified a total of 391 orthogroups significantly associated with aflatoxin production (Fig. 4). Of these 391, a total of 91 orthogroups were not annotated either with NCBI blast or InterProScan. There were 369 orthogroups (94.4%) from shell genome. Important orthologous gene clusters with significant association with aflatoxin production were listed in (Table 2). Surprisingly, of the 369 shell genes, there were 256 (69.4%) orthogroups which were not annotated and may be absent in the AF13 reference genome assembly. The new orthogroups significantly associated with aflatoxin production were potential intermediates in various aflatoxin biosynthetic pathways in *A. flavus*. There were 15 orthogroups from soft-core and seven from cloud genome significantly associated with aflatoxin production, including aspergilol biosynthesis cluster protein, aspyridones biosynthesis protein E (*apdE*), and imizoquin biosynthesis cluster protein D (*imqD*) (Supplementary Table 7).

**Table 2:** Pan-GWAS identified orthogroups associated with aflatoxin (ppb) production in *Aspergillus flavus*

Orthogroup^a^	-log10(p)^b^	Genome^c^	Length^d^	Annotation^e^
OG0012151	7.64	Shell	444	Aflatoxin biosynthesis protein R (*aflR*)
OG0012150	7.64	Shell	438	Aflatoxin biosynthesis protein S (*aflS*)
OG0011833	7.06	Shell	458	Fusaristatin A biosynthesis cluster protein
OG0000052	9.97	Soft-core	764	Aspyridones biosynthesis protein F
OG0012617	6.33	Shell Non-reference	493	Neosartiricin biosynthesis protein R
OG0012284	6.54	Shell	317	Cyclochlorotine biosynthesis protein O
OG0012070	7.16	Shell	534	Cytochrome P450 monooxygenase; Acurin A biosynthesis cluster protein (*acrF*)
OG0012018	6.27	Shell	508	Cytochrome P450 monooxygenase aflV; Aflatoxin biosynthesis protein (*aflV*)
OG0012566	7.12	Shell	470	Cytochrome P450 monooxygenase; AK-toxin biosynthesis protein 7 (*AKT7*)
OG0000168	6.55	Shell Non-reference	521	Cytochrome P450 monooxygenase aneD; Aculenes biosynthesis cluster protein D (*aneD*)
OG0010996	16.78	Soft-core	215	Cytochrome P450 monooxygenase apdE; Aspyridones biosynthesis protein E (*apdE*)
OG0013427	22.40	Shell Non-reference	341	Dehydrogenase azaJ; Azaphilone biosynthesis cluster protein azaJ (*azaJ*)
OG0011609	7.22	Shell Non-reference	345	Dehydrogenase RED2; T-toxin biosynthesis protein (RED2)
OG0012391	7.34	Shell	116	Demethylsterigmatocystin 6-O-methyltransferase; Aflatoxin biosynthesis protein O (*aflO*)
OG0012144	7.47	Shell	548	Efflux pump aflT; Aflatoxin biosynthesis protein T (*aflT*)
OG0013752	6.10	Shell Non-reference	261	Efflux pump atB; Terreic acid biosynthesis cluster protein B (*atB*)
OG0012168	6.89	Shell Non-reference	541	Efflux pump mlcE; Compactin biosynthesis protein E (*mlcE*)
OG0011998	6.55	Shell	651	Efflux pump radE; Radicicol biosynthesis cluster protein (*radE*)
OG0012554	7.92	Shell Non-reference	1,102	Efflux pump terG; Terrein biosynthesis cluster protein (*terG*)
OG0012658	6.81	Shell Non-reference	508	Efflux pump terG; Terrein biosynthesis cluster protein (*terG*)
OG0012155	9.12	Shell Non-reference	208	Enterobactin synthase component B; Enterobactin biosynthesis bifunctional protein (*EntB*)
OG0012119	7.64	Shell	1,737	Fatty acid synthase alpha subunit aflA; Aflatoxin biosynthesis protein A (*aflA*)
OG0012283	6.46	Shell Non-reference	636	Fumagillin dodecapentaenoate synthase; Fumagillin biosynthesis polyketide synthase
OG0014250	10.44	Shell Non-reference	220	Methyltransferase pytC; Pyranterreones biosynthesis cluster protein C (*pytC*)
OG0012525	6.62	Shell	330	MFS gliotoxin efflux transporter gliA; Gliotoxin biosynthesis protein A (*gliA*)
OG0012623	6.44	Shell	497	MFS transporter cpaT; Cyclopiazonic acid biosynthesis cluster protein T (*cpaT*)
OG0013082	7.78	Shell Non-reference	317	Notoamide biosynthesis activator; Notoamide biosynthesis cluster protein L (*NotL*)
OG0011985	6.13	Shell	643	Dehydrogenase patE; Patulin biosynthesis cluster protein E (*patE*)
OG0000472	6.52	Soft-core	720	Peptide transporter imqD; Imizoquin biosynthesis cluster protein D (*imqD*)
OG0012742	7.29	Shell Non-reference	707	Prenyltransferase phnF; Phenalenone biosynthesis cluster protein F (*phnF*)
OG0012565	7.81	Shell	251	Probable tetra hydroxynaphthalene reductase; Conidial pigment biosynthesis cluster
OG0012428	6.26	Shell	286	Aspercryptin biosynthesis cluster protein L (*atnL*)
OG0012853	17.19	Shell	726	Cyclochlorotine biosynthesis protein T (*cctT*)
OG0012547	6.95	Shell	1,060	Melleolides biosynthesis cluster protein
OG0012333	6.85	Shell Non-reference	599	Fusicoccin A biosynthetic gene clusters protein
OG0012630	6.81	Shell Non-reference	451	Satratoxin biosynthesis SC3 cluster protein 17 (*SAT17*)

^a^Orthogroups of AflaPan significantly associated with toxin production

^b^-log10 transformation of *p* values identified in Pan-GWAS analysis

^c^Genome (Core, soft-core, shell or cloud) of the orthogroups. Where, the new genes are denoted by suffix non-reference. For instance, shell non-reference means, the new orthogroups in the shell genome. Whereas, ‘Shell’ means the orthogroups already existing in previous reference genomes

^d^Protein sequence length of orthogroup

^e^Annotation of orthogroups

Among highly associated aflatoxin producing gene clusters there were cytochrome P450 monooxygenase (*apdE*, *acrF*, *aflV*, *AKT7*, *aneD*), ABC multidrug transporters (*atrB*, *atrD*, *MDR2*), aflatoxin biosynthesis regulatory protein R (*aflR*), aflatoxin biosynthesis protein S (*aflS*), demethylsterigmatocystin 6-O-methyltransferase (*aflO*), aflatoxin biosynthesis protein T (*aflT*), fatty acid synthase alpha subunit (*aflA*), beta-cyclopiazonate dehydrogenase, and fumagillin biosynthesis polyketide synthase. aflaoxin producing orthologous gene clusters homologous to aflatoxin producing genes in other fungi were also showed strong association with aflatoxin production in this AflaPan, for instance, terrein biosynthesis cluster protein (*terG*) from *A. terreus*, ABC multidrug transporter (*atrB*) from *A. nidulans*, Azaphilone biosynthesis cluster protein (*azaJ*) from *A. niger*, double-stranded DNA deaminase aflatoxin A from *Burkholderia cenocepacia*, glutathione S-transferase omega from *Saccharomyces cerevisiae*, and AM-toxin biosynthesis regulator 1 (*AMTR-1*) from *Alternaria alternata* (Supplementary Table 7).

## Discussion

Aflatoxin is detrimental to human health specially affecting liver and gall bladder in addition to problems of growth stunting and impaired development. The aflatoxin producing pathogen, *Aspergillus flavus*, has acquired large geographical diversity and broad host range over the time and sustained under varied climatic conditions. The aflatoxin contamination has been global food safety concern for humans and animals, many a times even leading to death. Isolated efforts have been made though for identifying the genomic regions and candidate genes controlling different aflatoxin resistance mechanisms [4], however, less efforts are being made to in depth understanding adoption of isolates specially under changing climate conditions. Nevertheless, the recently sequenced two reference-level genomes for two *A. flavus* isolates (isolate AF13 of mating type *MAT1-2* with high producer of aflatoxin and isolate NRRL3357 of mating type *MAT1-1* with a moderate producer) facilitated identification of 310 kb region being the major aflatoxin producing gene cluster [14]. As of now not much understanding has been developed on the extent of diversity among pathogens from different geographies and crops. Therefore, this comprehensive research with large number of sequenced genomes has been conducted to solve the puzzle of evolution of isolates, intensity of infection to plants and varied level of aflatoxin production under different climatic conditions. We collected and sequenced new set of 225 *A. flavus* isolates and also included 121 publicly available sequences for genome analysis. This is the maiden effort to develop pangenome of *A. flavus*, called AflaPan, and perform other genomic analysis using 346 genome sequences. The AflaPan had 17,855 unique orthologous gene clusters including 7,315 core genes (41%) and 10,540 accessory genes indicating significant genome diversity between *A. flavus* isolates. It is interesting to note that there were 5,994 orthologous gene clusters that had been not annotated in *A. flavus* AF13 and NRRL3357 reference genomes [14]. The pan-GWAS study using AflaPan identified 391 significant associated orthologous gene clusters for aflatoxin production, indicating the usefulness of AflaPan. It’s worthwhile noting that AflaPan represents a large diversity of 346 *A. flavus* genomes from 11 states of United States with aflatoxin contamination issues in various crops (Fig. 1). Although no such study has been reported for *A. flavus*, however, recently two pangenomes have been reported for related species *A. fumigatus* with 300 [19] and 260 [21] isolates. It is important to note that these two pangenomes are closed in nature similar to AflaPan indicating sufficient sample size for capturing complete species genome diversity.

The accessory genome is a variable portion of pangenome and higher accessory gene content indicates higher diversity among the individuals. AflaPan had large proportion of accessory genome (58.9%; 10,540 genes) (Fig. 3a) as compared to *A. fumigatus* (41% accessory genome; [19] and *Candida albicans* (mere 9% accessory genome; [18]. The high accessory genome content in *A. flavus* indicates the faster pace of genome evolution which might be its strength for its successful adaptation to different climatic conditions, geographies and host plants. This finding also signals warning to research community for much faster devising and implementing solutions to tackle the aflatoxin contamination problem.

Pangenomes can be classified as open pangenomes or closed pangenomes [59]. If the number of accessory and core genes of a pangenome are stable and not increasing after adding an optimum number of genomes, then it is referred to as a closed pangenome. In contrast, if the number of accessory and core genes of a pangenome are substantially increasing upon addition of individual genomes then it is referred as an open pangenome. An open pangenome can be substantially improved by sequencing more individuals resulting in more accessory and core genes being included in the existing pangenome [60]. In AflaPan, the number of accessory and core genes were not substantially increased after adding 300 *A. flavus* genomes. Therefore, AflaPan is a closed pangenome, and effectively captured the overall genomic diversity of the *A. flavus* species. In contrast, the pangenome of the wheat fungal pathogen *Zymoseptoria tritici* was developed with only 19 isolates which can be improved further by including more isolates [61]. Similarly, recently another open pangenome of an onion bacterial pathogen *Pantoea ananatis* was developed using 81 *P. ananatis* genomes which showed an increase of 50 new genes with addition of each isolate [62]. While exploring possibilities for genome expansion or horizontal gene transfer [63] in *A. flavus* genomes, the isolates from soil samples had larger genomes in comparison with isolates collected from infected corn tissues. The expanded soil isolates’ genomes included non-reference aflatoxin producing gene clusters identified in the Pan-GWAS analysis using AflaPan.

One important objective behind developing AflaPan was to conduct Pan-GWAS study and to identify unreported aflatoxin producing gene clusters. In this study, the newly sequenced isolates of 225 were used for phenotyping aflatoxin production and Pan-GWAS analysis to identify the gene clusters associated with aflatoxin production. Pan-GWAS analysis for aflatoxin production, using a presence/absence matrix based on 17,855 pan-genes across 225 isolates, identified 391 pan-genes significantly associated with aflatoxin production. Among them, 369 pan-genes (94.4%) were located in the shell genome of AflaPan. Our findings suggest that the shell genome of AflaPan is enriched with aflatoxin producing gene clusters. Shell genome includes pan-genes which are present in 95% of the total individuals (Supplementary Table 7). Interestingly, a total of 256 new pan-genes which are not annotated in present reference genomes NRRL3357 and AF13 were significantly associated with aflatoxin production. These 256 pan-genes could be important targets for developing aflatoxin mitigation strategies using gene silencing or genome editing approaches. Earlier, Pan-GWAS has been conducted in several fungi as well as bacterial pathogens to identify causal genes. For instance, Pan-GWAS in *Pantoea ananatis* pangenome identified 14 strongly associated genes in HiVir/PASVIL clusters responsible for pathogenicity in onion [61]. Recently, human pathogen *A. fumigatus* has developed resistance against fungicide triazoles. Pan-genome wide association analysis using *A. fumigatus* pangenome of 300 isolates identified 12 genes associated with triazole resistance [19]. Another, Pan-GWAS using *A. fumigatus* pangenome of 218 isolates identified azole drug resistant genes in ergosterol biosynthetic pathway [20].

Two genes in *A. flavus*, *aflS* and *aflR*, mainly regulate the aflatoxin biosynthesis pathway [64], with *aflR* encoding a DNA-binding binuclear zinc cluster (Zn(II)_2_Cys_6_) transcription factor required for expression of a majority of structural genes in aflatoxin biosynthesis pathway [65]. Here, Pan-GWAS analysis using AflaPan identified significant association with *aflR* with -log10(*p*) = 7.6. *aflS* has also been shown to be essential for aflatoxin biosynthesis and is required for activation of *aflR* [66]. Disruption of *aflS* in *A. parasiticus* resulted in lower expression of some AFB1 producing genes, such as *aflC*, *aflD*, *aflM* and *aflP* and reduced aflatoxin levels [67]. It is also observed that *aflS* is strongly associated with aflatoxin production with -log10(*p*) = 7.64. In addition, there are several aflatoxin biosynthesis genes including *aflV*, *aflT*, *aflA* and *aflO* which showed strong associations with aflatoxin production according to the Pan-GWAS analysis (Table 2). *aflV* showed to be associated with aflatoxin production with -log10(*p*) = 6.3. *aflV* encodes cytochrome P450 monoxygenase (*cypX*) has been shown to catalyze the reaction from averufin (AVR) to hydroxyversicolorone (HVN) and HVN further involves in aflatoxin biosynthesis [68]. It is reported that *aflT* is not directly linked with aflatoxin biosynthesis, though it is present in aflatoxin producing gene cluster [69]. However, Pan-GWAS analysis indicated that *aflT* is significantly associated with aflatoxin production with -log10(*p*) = 7.5. The *fas* (fatty acid synthases) genes, namely *aflA* also referred as ‘*fas-2*’ and *aflB* referred as ‘*fas-1*’ produces α and β sub-units, respectively, both subunits transform the hexanoate units into a polyketide structure in aflatoxin biosynthesis pathway [70]. In this study, *aflA* (*fas-2*) showed strong association with aflatoxins with -log10(*p*) = 7.6. The *aflO* (*omtB*) homolog in *A. nidulans* required for conversion of demethylsterigmatocystin (DMST) to sterigmatocystin (ST) in *A. nidulans* [71].

Pan-GWAS analysis showed significant association with previously reported aflatoxin producing gene clusters [72] such as O-methyltransferase A (*OmtA/AflP*) and versicolorin dehydrogenase /ketoreductase (*Ver1/AflM*) with –log10(*p*) value of 7.4 and 6.8, respectively. *OmtA/AflP* and *Ver1/AflM* had been targeted for host-induced gene silencing (HIGS) in peanuts resulting in reduced aflatoxin contamination [73]. Moreover, *Ver1/AflM* was also targeted for host induced gene silencing in maize [74].

*A. flavus* is also a reservoir for important secondary metabolites [75]. In this study, we investigated 10 secondary metabolites and their associated genes, investigating the importance of AflaPan genome. Interestingly, in AflaPan, there were 165 pan-genes annotated to associate with production of 10 important secondary metabolites (Supplementary Table 6). Recently, pan-secondary metabolome based on 94 *A. flavus* isolates identified 7,821 biosynthetic gene clusters (BGCs) responsible for production of variety of metabolites [11]. The secondary metabolite producing gene clusters for these 10 metabolites were identified in core as well as accessory genomes of this AflaPan. Kojic acid (KA) has commercial use in industries including cosmetics, especially in skin care product to prevent the exposure to UV radiations [56]. In AflaPan, three pan-genes were identified as Kojic acid biosynthesis genes. In addition, the pan-genes also associated with the production of aflavarin, aflatoxin, asparasone, cyclopiazonic acid, ditryptophenaline, leporins, piperazines, and ustiloxin.

In summary, this study provides complete pangenome framework for the species of *Aspergillus flavus* along with associated genes for pathogen survival and aflatoxin production. This pangenome can be used for performing further studies on target traits as well as developing required diagnostic genotyping panel for isolate identification. Furthermore, the information generated through this study can also be used for identifying individuals from different diversity clusters for developing individual genome assemblies leading development of pangenome reference. Most importantly, the newly identified aflatoxin producing gene clusters will be a new source for seeking aflatoxin mitigation strategies and needs new attention in research.

### Electronic supplementary material

Below is the link to the electronic supplementary material.

Supplementary Material 1

Supplementary Material 2

## Data Availability

Analyzed data on sequencing statistics, assembly statistics, SNP statistics and Pan-GWAS analysis are provided in the attached supplementary files. The newly developed genome assemblies for 225 isolates used in this study are available at https://zenodo.org/deposit/7615243. Associated raw sequencing data for each isolate are available through National Center for Biotechnology Information (NCBI) - Sequence Read Archive (SRA) with Bioproject ID PRJNA915632. Fungal isolates are available upon request by contacting corresponding author. The 121 isolates from public data can be requested from corresponding authors.

## Abbreviations

AVR: averufin
AFB1: aflatoxin B1
AFB2: aflatoxin B2
AFG1: aflatoxin G1
AFG2: aflatoxin G2
AMTR-1: AM-toxin biosynthesis regulator 1
BGCs: biosynthetic gene clusters
CPA: cyclopiazonic acid
DMST: demethylsterigmatocystin
HIGS: host-induced gene silencing
HVN: hydroxyversicolorone
iTOL: interactive tree of life
KA: kojic acid
LD: linkage disequilibrium
MLM: mixed-linear-model
MDRB: Modified Dichloran Rose Bengal
NCBI-SRA: National Center for Biotechnology Information - Sequence Read Archive
PAV: presence/absence variance matrix
qRT-PCR: Quantitative real time polymerase chain reaction
SNVs: single nucleotide variants
ST: sterigmatocystin
TPCs: toxins producing gene clusters
YES: yeast extract sucrose
