# Supplementary material for: Aspergillus flavus pangenome (AflaPan) uncovers novel aflatoxin and secondary metabolite associated gene clusters
Source: BMC Plant Biol. 2024 May 1;24:354. doi: 10.1186/s12870-024-04950-8 (PMC11061970; doi:10.1186/s12870-024-04950-8)
Supplement: Supplementary file 1 — Supplementary Material 1 [file 12870_2024_4950_MOESM1_ESM.docx]

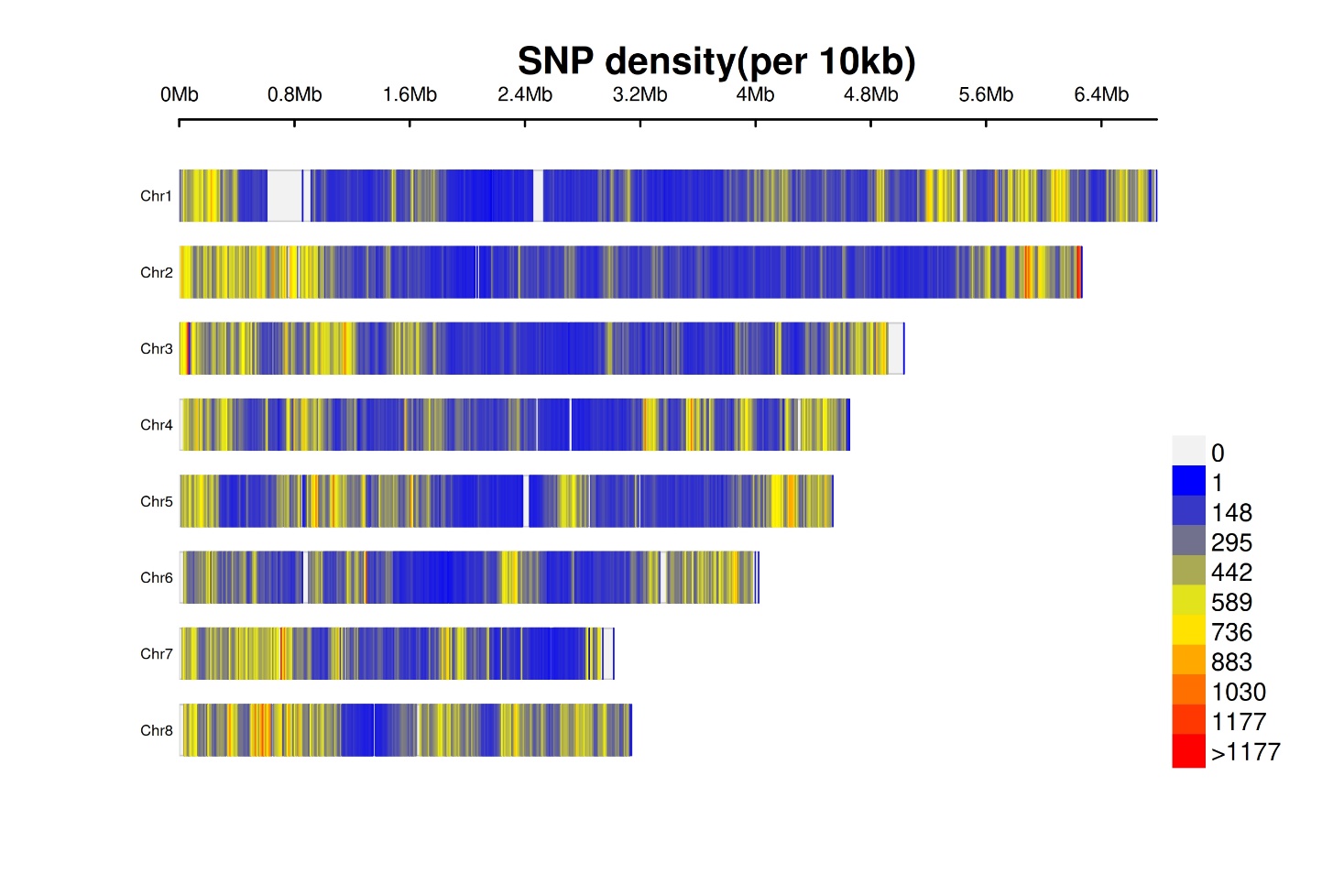


**Supplementary Figure 1.** Density of single nucleotide variants called using whole genome resequencing data of 346 *A. flavus* isolates


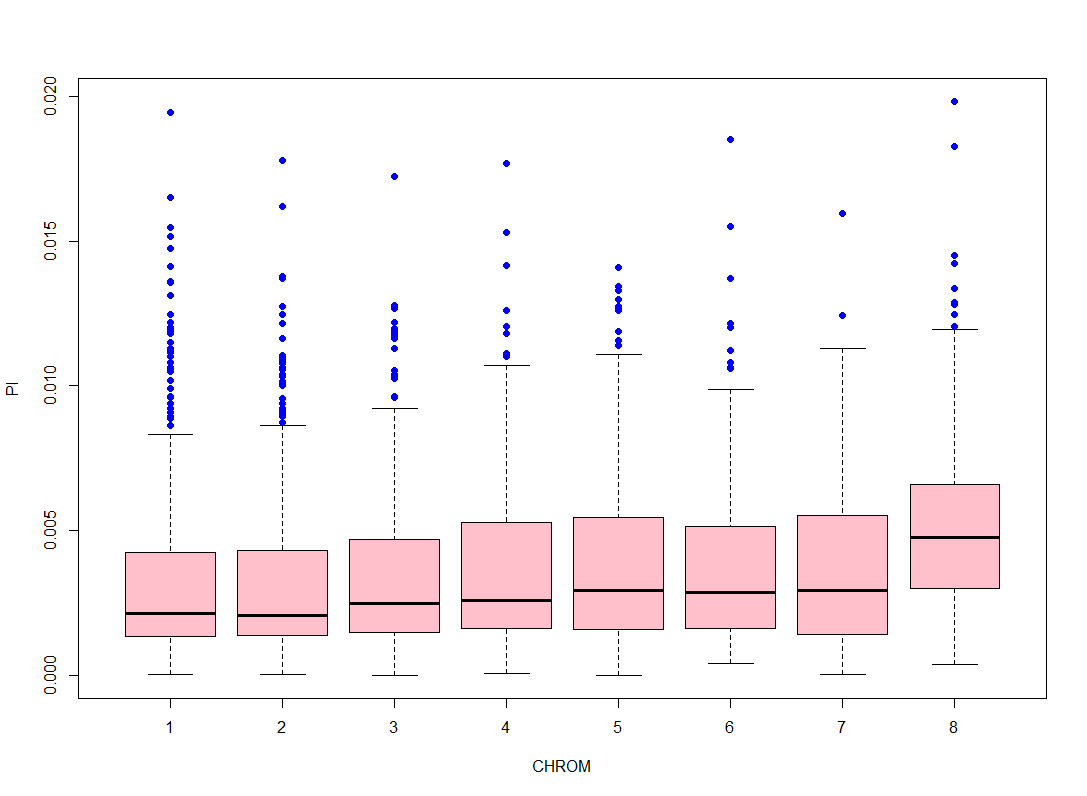
**Supplementary Figure 2.** Chromosome wise nucleotide diversity (*π*) based on SNVs across 346 isolates calculated using 10000bp window. Highest mean nucleotide diversity (*π*) was identified on chromosome 8.


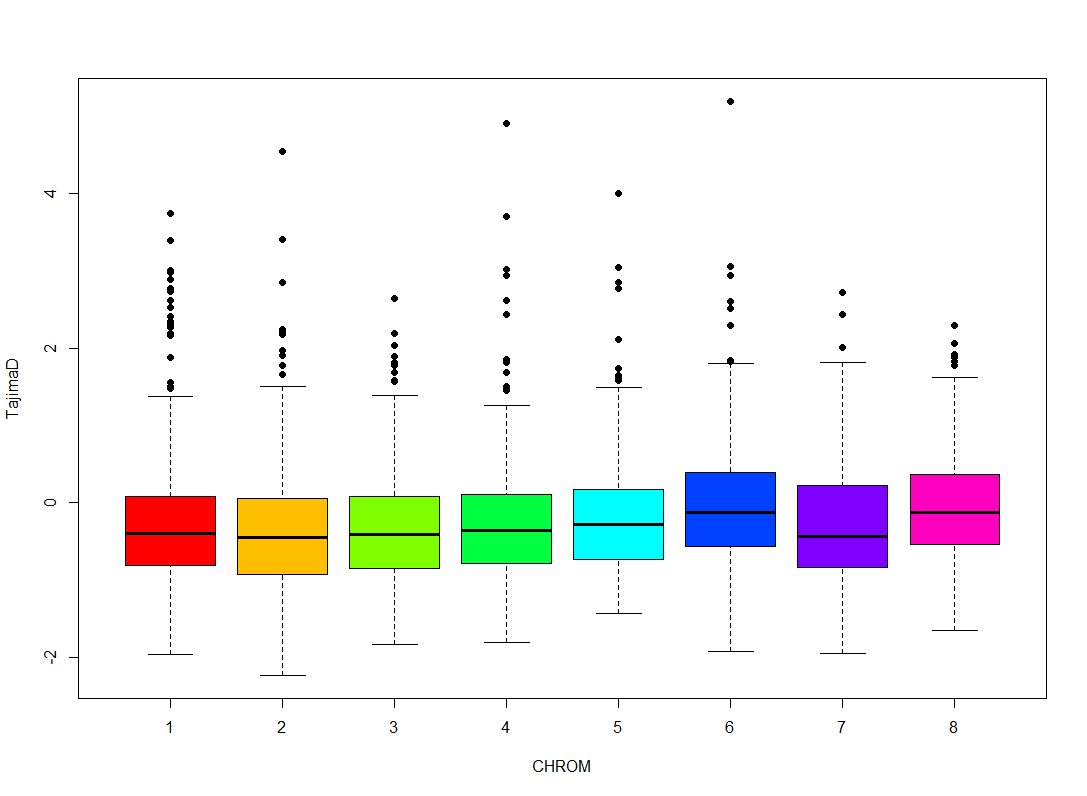


**Supplementary Figure 3.** Chromosome wise Tajimas’s D diversity index calculated using SNVs across 346 isolates in a 10000bp window.
